# Composite Membranes Derived from Cellulose and Lignin Sulfonate for Selective Separations and Antifouling Aspects

**DOI:** 10.3390/nano9060867

**Published:** 2019-06-07

**Authors:** Andrew Colburn, Ronald J. Vogler, Aum Patel, Mariah Bezold, John Craven, Chunqing Liu, Dibakar Bhattacharyya

**Affiliations:** 1 Department of Chemical and Materials Engineering, University of Kentucky, Lexington, KY 40506, USA; andrew.colburn@uky.edu (A.C.); voglerronald@uky.edu (R.J.V.); aum.patel@uky.edu (A.P.); mariah.bezold@uky.edu (M.B.); johndcraven@gmail.com (J.C.); 2 R&D Department, Honeywell UOP, Des Plaines, IL 60016, USA; Chunqing.Liu@Honeywell.com

**Keywords:** nanocomposite, ionic liquid, selective separation, water application

## Abstract

Cellulose-based membrane materials allow for separations in both aqueous solutions and organic solvents. The addition of nanocomposites into cellulose structure is facilitated through steric interaction and strong hydrogen bonding with the hydroxy groups present within cellulose. An ionic liquid, 1-ethyl-3-methylimidazolium acetate, was used as a solvent for microcrystalline cellulose to incorporate graphene oxide quantum dots into cellulose membranes. In this work, other composite materials such as, iron oxide nanoparticles, polyacrylic acid, and lignin sulfonate have all been uniformly incorporated into cellulose membranes utilizing ionic liquid cosolvents. Integration of iron into cellulose membranes resulted in high selectivity (>99%) of neutral red and methylene blue model dyes separation over salts with a high permeability of 17 LMH/bar. With non-aqueous (alcohol) solvent, iron–cellulose composite membranes become less selective and more permeable, suggesting the interaction of iron ions cellulose OH groups plays a major role in pore structure. Polyacrylic acid was integrated into cellulose membranes to add pH responsive behavior and capacity for metal ion capture. Calcium capture of 55 mg Ca^2+^/g membrane was observed for PAA-cellulose membranes. Lignin sulfonate was also incorporated into cellulose membranes to add strong negative charge and a steric barrier to enhance antifouling behavior. Lignin sulfonate was also functionalized on the commercial DOW NF270 nanofiltration membranes via esterification of hydroxy groups with carboxyl group present on the membrane surface. Antifouling behavior was observed for both lignin-cellulose composite and commercial membranes functionalized with lignin. Up to 90% recovery of water flux after repeated cycles of fouling was observed for both types of lignin functionalized membranes while flux recovery of up to 60% was observed for unmodified membranes.

## 1. Introduction

Cellulose is the most abundant biopolymer on the earth. The structural significance of cellulose toward life on Earth is profound, as it makes up most of the cell wall in plants, providing structural support. Cellulose within the cell wall of plants arranges itself in a mesoporous structure to sterically prevent enzymatic decomposition [1]. The robust characteristics of cellulose fibers have been utilized by humans for millennia. The use of cellulose fibers as a membrane-like material arguably began when humans first began making textiles out of cotton in the 6th millennium BC in modern day India and Pakistan [2]. Cellulose fibers remain an effective material for physical size-based separations of particulates such as algae clusters that act as carriers for cholera [3,4]. Beyond particle separation, the polymer network of cellulose materials has been investigated for the separation of smaller organic molecules. Transport of solutes through cellulose membranes has been of interest in the scientific community for many years. Dating back to the 1950s, hindered diffusion of small organic molecules in aqueous solution through cellulose materials such as cellophane and sausage casings were studied [5]. Cellulose-derived polymers such as cellulose acetate have been widely used for membrane making, but the modification required to enhance solubility in commercial solvents reduces robustness of membrane during filtration of organic solvents [6].

The strong hydrogen bonding between cellulose chains poses challenges for dissolution and processing of cellulosic material. New solvent developments have enabled synthesis of cellulose membranes without modification of the chemical structure of cellulose. Regeneration of cellulose with solvents such as N-methylmorpholine-N-oxide or basic conditions is being implemented to create selective membranes [7,8,9]. Ionic liquids are being investigated as a new solvent for regeneration of cellulose for membrane synthesis [10,11]. Membranes utilizing ionic liquid as a solvent have been shown to perform in the ultrafiltration or nanofiltration regimes. This same ionic liquid approach was used to spin cellulose hollow fibers [12]. Cellulose membranes prepared using ionic liquid have been shown to be highly selective for organic dyes, rejecting 94% of Bromothymol Blue [13]. Cellulose membranes have been shown to be effective at separating negatively charged oil emulsions from water while maintaining minimum fouling [14].

Composite materials have been integrated into polymer membranes to enhance properties and performance. Cellulose is a particularly interesting polymer for the integration of composite materials through hydrogen bonding via the hydroxy groups, which aid steric entanglement in nanocomposite retention. This allows for retention of composite materials that strongly interact with hydroxy groups such as layered double hydroxide [15]. Incorporation of graphene quantum dots into cellulose via ionic liquid was demonstrated to add negative charge and improve the selectivity of model dyes [16].

Beyond modifying membrane pore structure, the development of composite and blended materials brings additional properties that improve membrane performance and expand membrane application. Polyacids, particularly polyacrylic acid (PAA) have been incorporated into membrane supports to allow for strong negative charge, metal capture, and pH responsive behavior. PAA has been crosslinked within a PAA membrane pore to functionalize the pore with carboxyl groups for metal capture and in-situ nanoparticle functionalization [17]. PAA has also been grafted onto cellulose nanofiber mats for use in the capture of heavy metals [18]. Lignin, a complex plant-derived polymer, is another material that has capacity for heavy metal capture [19]. Due to the abundance of hydrophilic groups, lignin can be incorporated as a composite material with other polymers. Composite SPEEK/lignin membranes have been demonstrated to create a tighter pore structure than conventional Nafion membranes while allowing for enhanced proton transport [20].

The objective in this work was to further expand on our previous research studying cellulose graphene oxide quantum dots (GQD) membranes into other composite materials to further improve membrane performance and demonstrate flexibility of this technique for membrane development. Iron (III), polyacrylic acid, and lignin sulfonate were all investigated as composite materials for integration within the cellulose membrane domain. Membrane permeability and selectivity was studied for each composite type. Other useful properties unique to each composite material were observed such as metal capture and antifouling properties. Lignin sulfonate was also functionalized onto the surface of commercial NF membrane (NF270) to demonstrate antifouling behavior of the functionalized membrane surface through the creation of strong acid sulfonate groups on the surface.

## 2. Materials and Methods

### 2.1. Materials

1-ethyl-3-methylimidazolium acetate (EMIMAc, HPLC grade) was purchased from Sigma Aldrich (St. Louis, MO, USA. Avicel^®^ PH-101 microcrystalline cellulose (50 µm, cotton linter source) was purchased from Sigma Aldrich. Nonwoven polyester backing material from Solecta Membranes was used as a support for membrane formation. Blue dextran (MW: 5000 Da; 10,000 Da) were purchased from Sigma Aldrich for use in membrane pore size characterization. Solutes used in selectivity studies can be seen in Table 1. Methylene Blue and Neutral Red (Sigma Aldrich) were used as model dies to study rejection of molecules <1000 Da. A model dimer (2-(2-Methoxyphenoxy)-1-(4-methoxyphenyl)ethanol) was provided by Dr. Mark Crocker’s lab in the Center for Applied Energy Research. Ferric chloride (Fisher Scientific, Hanover Park, IL, USA) was used as an iron (III) source in composite membrane synthesis. Lignosulfonic acid sodium salt was purchased from Beantown Chemical LLC, Hudson, NH, USA. as a lignin sulfonate source. Humic acid (technical grade) and bovine serum albumin were purchased from Sigma Aldrich for antifouling study. Na2SO4 (1000 mg/L Fisher Scientific) was used to characterized nanofiltration membrane performance. The country of origin for all membranes and chemicals use was the United States of America.

### 2.2. Cellulose Composite Membranes

Three types of Cellulose composite membranes were studied: cellulose iron, cellulose PAA, and cellulose lignin sulfonate composite membranes. A summary of the composition of the various membranes can be seen in Table 2. Control membranes of 10 wt% cellulose were also studied. All membranes were created using 1-ethyl-3-methylimidazolium as a solvent. The desired amount of composite material was dispersed into the ionic liquid at 80 °C for one hour. This is to ensure full dispersion of the composite material in the ionic liquid before dissolution of cellulose increases the casting solution viscosity. After composite material dispersion, 5–10 wt% cellulose was added into the casting solution and physically mixed in then dissolved at 80 °C for 8 to 24 h until the cellulose was completely dissolved.

Membranes were cast on nonwoven fiber backing. Polyester support material was affixed to a glass plate using tape. The casting solution was poured directly onto the backing at 80 °C and cast directly onto the polyester backing using a doctor blade set to 150 μm. The polyester backing was then submerged in a water gelation bath for 10 min to allow time for membrane formation. The resulting membrane was stored in DI water at a temperature of 4 °C until use.

### 2.3. Zeta Potential Characterization

Zeta potential of cellulose and GQD cellulose membranes was measured by an Anton Paar Surpass 1 Electrokinetic Analyzer, Ashland, VA, USA. The adjustable gap cell was used with a 100 µm gap and 0.01 M KCl electrolyte solution. Acid titration was done with 0.01 M HCl. A 400 mBar pressure difference was used for all measurements.

### 2.4. Contact Angle Characterization

The contact angle for deionized ultrafiltered water was measured using the Kruss DSA 100, Matthews, NC, USA. Captive bubble method was used to determine contact angle do to water absorption in the cellulose membranes and to prevent deformation of surface structure during drying. At least 3 spots per membrane sample were analyzed to correct for any variance in surface morphology.

### 2.5. Membrane Performance

Membrane performance was characterized by using the Sterlitech HP4750 stirred cell to perform convective studies. Water permeability was determined for each membrane by measuring the volumetric flux of deionized ultrafiltered (DIUF) water at 1.4, 2.76, and 4.14 bar respectively. Methylene blue (5 mg/L) and neutral red (5 mg/L), as well as various molecular weights (5 kDa and 10 kD at concentrations of 100 mg/L) of Blue Dextran, were filtered through the membrane. The permeate was collected and dye concentration for the feed, permeate, and remaining retentate was analyzed using the VWR UV-6300PC Spectrophotometer.

For cellulose–lignin composite membranes, antifouling properties were analyzed by testing permeability of 100 mg/L humic acid solution at pH of 5.6 in a crossflow system.

### 2.6. Divalent Ion Capture by Cellulose-PAA Membranes

Ca^2+^ capture in cellulose PAA composite membranes was carried out following the procedure used by Islam et al. [17]. The cellulose PAA membrane was added to a Sterlitech HP4750 filtration cell and soaked in about 110 mL of DI water at a pH of 10. After soaking, about 15 mL of fresh DI water (pH » 4.5–5.5) was passed through the membrane and the pH of the permeate was verified to be 7 or higher. For the Ca^2+^ capture, an aqueous CaCl_2_·2H_2_O solution (»1.79 mM, pH = 6.5–7) was prepared with non-deoxygenated, DI water and a 10-mL sample of this solution was taken. To capture Ca^2+^, about 200 mL of fresh solution was passed through the membrane in 50-mL increments using pressures mostly in the range of 0.28–0.62 bar. At the end of each increment, a 10-mL sample of the collected permeate was taken and the rest of the permeate was disposed of before continuing the filtration.

Ca^2+^ captured was quantified by inductively coupled plasma optical emission spectroscopy. Ca^2+^ captured within the membrane case measured and located using energy-dispersive X-ray spectroscopy.

### 2.7. Lignin Sulfonate Functionalized Nanofiltration Membrane

Functionalized membranes were created by placing a 40 cm^2^ area of NF270 into a circular metal cell, an O-ring is typically found on these cells at the base to create a seal. A 10 wt% LS (lignin sulfonate) solution in water was poured over a 40 cm^2^ area NF270 nanofiltration membrane. Sufficient volume of lignin sulfonate was applied to cover the entire membrane surface. The cell was then placed in an oven at 90 °C for approximately 2 h. After taking the functionalized membrane out of the cell, it was then thoroughly rinsed with DI water to remove residual LS that may not have bonded to the membrane. LS presence on the membrane surface can be visually confirmed by light brown tint on the membrane surface.

A crossflow apparatus allowed for testing the anti-fouling properties of both the functionalized and unfunctionalized membrane. The cross-flow apparatus was run at a flowrate of approximately 1.5 liters/min for both the equilibration stage, fouling stage, and tangential washing stage. Before any anti-fouling testing could be done, the membrane was precompacted at 10.4 bar with deionized water to equilibrate the membrane before the fouling agent. After this equilibration period, a bovine albumin serum (BSA) solution was run through the apparatus and volume of permeate measured. After 30 min into the fouling stage, the membrane surface was rinsed with deionized water (pH = 5.6) for 10 min. Na_2_SO_4_ (1000 mg/L Fisher Scientific, Hanover Park, IL, USA) rejection was also determined in the crossflow cell at 10.4 bar.

### 2.8. Bacteria Fouling Studies

R. palustris strain CGA009 (ATCC BAA-98) was purchased from ATCC (American Type Culture Collection, NY, NY, USA). Solid media cultures were isolated on tryptic soy broth agar plates. Liquid cultures were pre-grown in tryptic soy broth purchased from Hardy Diagnostics, Santa maria, CA, USA, which contains (g L^−1^) casein peptone, 17; soy peptone, 3; NaCl, 5; K_2_HPO_4_, 2.5; Dextrose, 2.5. Pre-grown liquid cultures were concentrated by centrifugation at 2500 rpm for 5 min and washed 3 times with minimal media to use as an inoculant.

R. palustris adhered to membranes were grown using a modified minimal media [21] that contained (g L−1) Na_2_HPO_4_, 6.8; KH_2_PO_4_, 2.9; NaCl, 1.3; MgSO_4_ 7H_2_O, 0.4; CaCl_2_ 2H_2_O, 0.075; thiamine hydrochloride 0.001. Trace elements were provided by adding 10 mL L^−1^ of a solution containing (g L^−1^) FeCl_3_ 6H_2_O, 1.66; ZnCl_2_, 0.17; MnCl_2_, 0.06; CoCl_2_ 6H_2_O, 0.06; CuCl_2_ 2H_2_O, 0.04; CaCl_2_ 2H_2_O, 0.73; and Na_2_MoO_4_ 2H_2_O, 0.06. sodium glutamate (3.5–7 mM), and acetate (70 mM) were utilized as primary nitrogen and carbon sources.

Solutions of minimal media were diluted 1:10 with phosphate buffer (pH~7.2) for inoculation on the membrane surface. Inoculation of cellulose membranes was carried out by convectively passing 15 mL of the diluted media through the membrane at 1.4 bar in a stirred membrane cell. After inoculation the membranes were removed from the cell and submerged in minimal growth media in the absence of light for 10 days to allow some time for bacterial growth. The overall goal was to simulate bacteria deposition and growth on the membrane surface over long-term operation.

Bacteria adhered membranes were chemically fixed [22] prior to critical point drying by dosing growth media containing an inoculated membrane with glutaraldehyde (50% from) to bring the solution to 2.5% glutaraldehyde, and left to sit for 2 h at 25 °C. The media slowly replaced with ethanol by removing media and adding ethanol to bring the ethanol concentration up to 25%, 50%, 75%, 85%, and 96%, leaving the solution to rest for 1 h between adding ethanol.

## 3. Results and Discussion

### 3.1. Summary of Membranes

A summary of composite membrane compositions and properties is given in Table 2. Permeability of all composite membrane was shown to be improved over unmodified cellulose membranes. Iron was the only composite material demonstrated to improve the selectivity of cellulose membranes for small molecules. There are many factors that impact membrane selectivity, only a few of which this work will address, but casting viscosity and wt% may be one property which can be better optimized to improve membrane performance. The focus on this work is to highlight the possibilities of incorporating composite materials into cellulose membranes and the unique benefits composite materials bring to membrane performance.

### 3.2. Iron Cellulose Composite Membranes

Prior work integrating graphene oxide quantum dots (GQDs) showed potential for suitably small nanomaterials to increase selectivity via improving the structure of the selective cellulose network by linking cellulose chains via hydrogen bonding. Nanocomposite membranes prepared with GQD were found to show over 80% rejection of methylene blue and greater than 95% rejection of 5000 kDa blue dextran while maintaining permeability over 12 LMH/bar. Due to difficulties in purification of GQD, it is desired to investigate other composite materials that can be incorporated at higher concentrations while maintaining controlled particle size.

**Figure 1:**
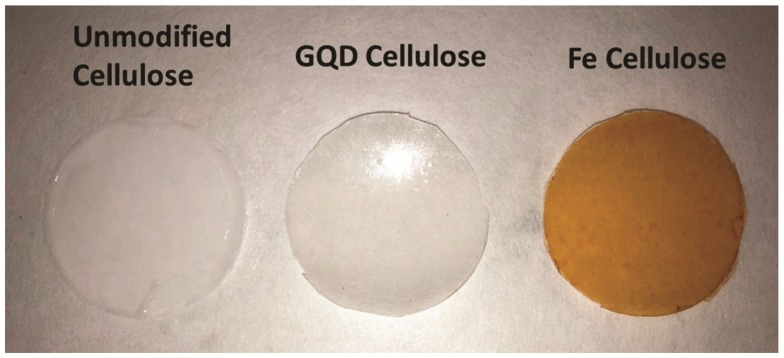
Unmodified cellulose, graphene oxide quantum dots (GQD) cellulose, and iron cellulose composite membranes.

Iron was readily incorporated as a composite material into the cellulose membrane domain, as FeCl_3_ is highly dispersible in ionic liquids. Iron is well known to interact strongly with cellulose and to bind to cellulose chains. This interaction along with steric effects ensure iron is retained within the membrane structure after phase inversion. A clear sign of the presence of iron within the membrane can been seen by the orange color the iron brings to the normally translucent cellulose membrane. This can be seen in Figure 1. This effect has also been observed in our prior studies with graphene oxide quantum dots as nanocomposites.

**Figure 2:**
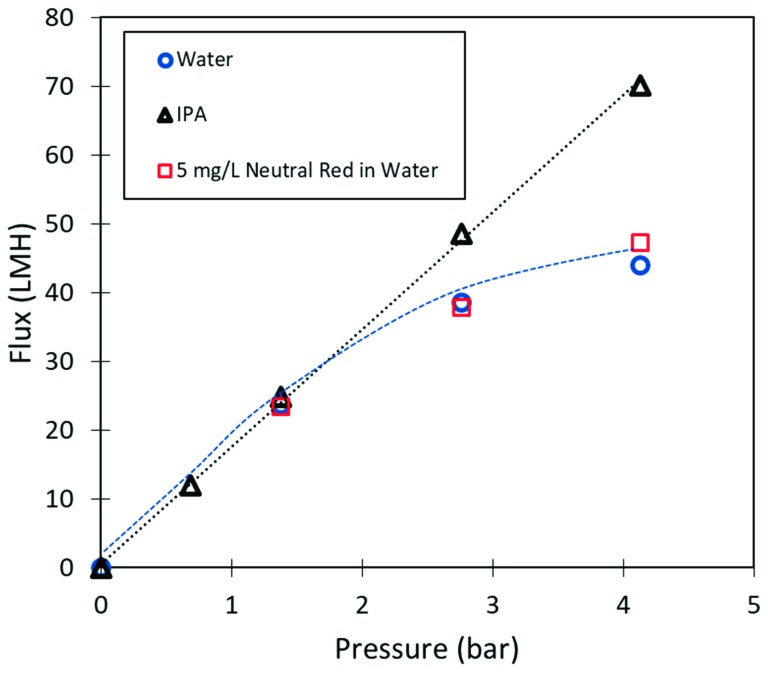
Flux (LMH, liter/m^2^-bar) vs. pressure (bar) behavior for iron cellulose composite membranes in water, isopropanol (IPA), and neutral red in water.

While uses of iron in composite materials and membrane platforms is well known, the main interest in this work was to understand if iron interaction with cellulose in the membrane effects selectivity behavior of the membrane. Previous study of cellulose composite membranes has suggested that selectivity behavior is largely due to a dense amorphous polymer layer that comprises the top 100–200 nm of the membrane. To better understand how iron and cellulose might be interact in the amorphous selective layer, the pressure dependent flux behavior of the membrane was studied in water and IPA solvents. As seen in Figure 2, water and isopropanol permeability behavior is unique when compared to expected behavior for cellulose based membranes. Water flux plateaued off at higher pressures, as previously observed in our studies of GQD cellulose composite membranes. Permeability of the iron cellulose membranes was within standard deviation of previously studied cellulose membranes, despite the iron composite casting solution having half the concentration of cellulose, as compared to previously studied cellulose membranes. Ordinarily reduced concentration of cellulose in the casting solution results in higher permeability. It is observed that the 4 wt.% of iron (III) chloride in the casting solution contributes to the development of a dense layer at lower cellulose concentrations. It is important to note that the 5 mg/L neutral red solution was permeated through the membrane after IPA passage, demonstrating that the flux response is reversible with solvent exchange.

**Figure 3:**
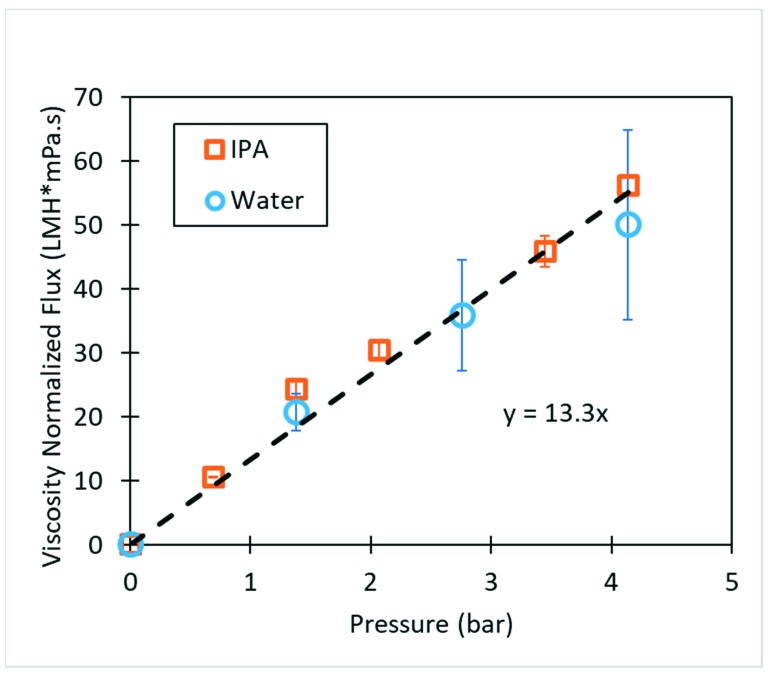
Viscosity corrected flux vs. pressure for unmodified cellulose membrane (10 wt. %). Temp 25 °C.

Further investigation of pressure dependent isopropanol flux gave unexpected results. Despite the viscosity of IPA being roughly double that of water, the permeability remains the same. Isopropanol permeability had previously been studied in cellulose membranes as seen in Figure 3 to observe the effect of the polar solvent on membrane stability. When corrected for viscosity, isopropanol and water flux behavior follow the same linear trend. This strongly suggests the membrane surface does not swell when in contact with isopropanol. IPA flux was higher than what would be expected when observing water permeability. This suggests that the membrane becomes more permeable when exposed to isopropanol.

Flux behavior was also found to be reversible as solvent was varied between water and isopropanol. No significant concentrations of iron ions were found to leach out of the membrane beyond the initial formation of the membrane via phase inversion. Considering lack of evidence of significant leaching and reversible flux response during solvent exchange, iron cellulose composite membranes can be assumed to be reasonably stable in neutral or basic conditions. Water was kept above pH 5 for all iron cellulose composite membrane experiments. At acidic pH complete ionization of iron oxide may result in increased leaching of iron from the membrane domain.

**Figure 4:**
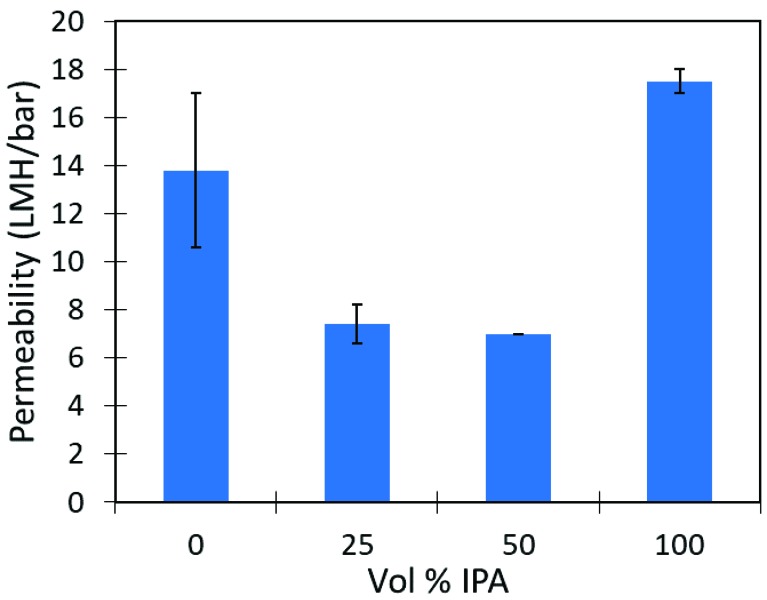
Volumetric permeability of total solvent mixture as volume % of isopropanol is varied in iron cellulose composite membranes. Remaining volume % water.

Interestingly, water permeability was decreased at solvent mixtures of 25:75 and 50:50 isopropanol:water as compared to either pure water or pure isopropanol. The permeability at different solvent concentrations can be seen in Figure 4. Strong water interaction within the cellulose domain may provide a barrier for isopropanol diffusion into the membrane domain. Mao et al. has observed that flux through cellulose membranes declined as isopropanol concentration increased during pervaporation operation [9]. Membrane permeability increased for pure isopropanol solvent, as iron is unable to be ionized after the 100% isopropanol solution removes residual water in the membrane. Absence of Fe^3+^ ions decreases interaction between cellulose chains, which may be responsible for the higher permeability in pure solvent conditions. When the membrane is rehydrated, more ionization of iron particles to Fe^3+^ occurs and the selective layer becomes denser. This behavior could be of great interest for applications of membrane cleaning or desorption of contaminants from the membrane surface.

**Figure 5:**
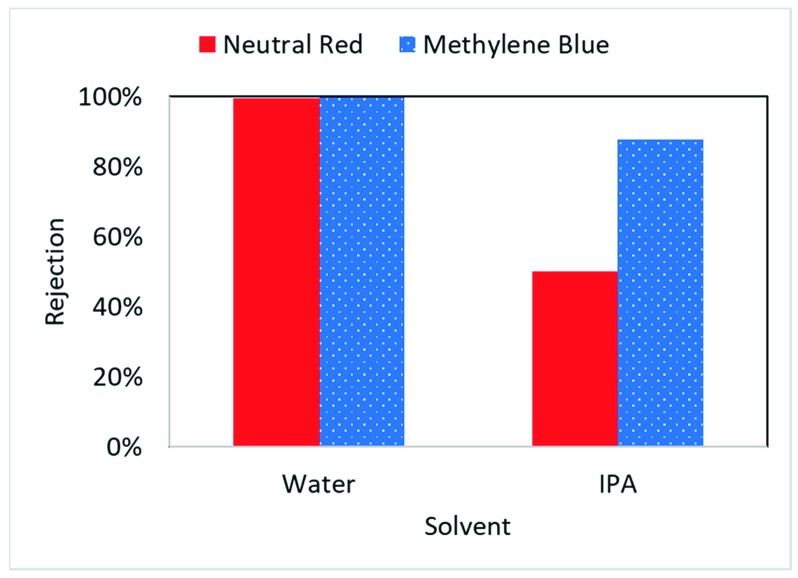
Dye rejection in iron cellulose composite membrane in water and isopropanol solvent.

Neutral red (~289 Da) and methylene blue (~320 Da) were completely rejected (>99%) during filtration through the membrane using DI water as a solvent. As seen in Figure 5 rejection decreased in isopropanol which is to be expected due as hydrophilic interaction decreases in isopropanol. The increase of membrane permeability suggests the dense selective layer becomes more permeable.

**Figure 6:**
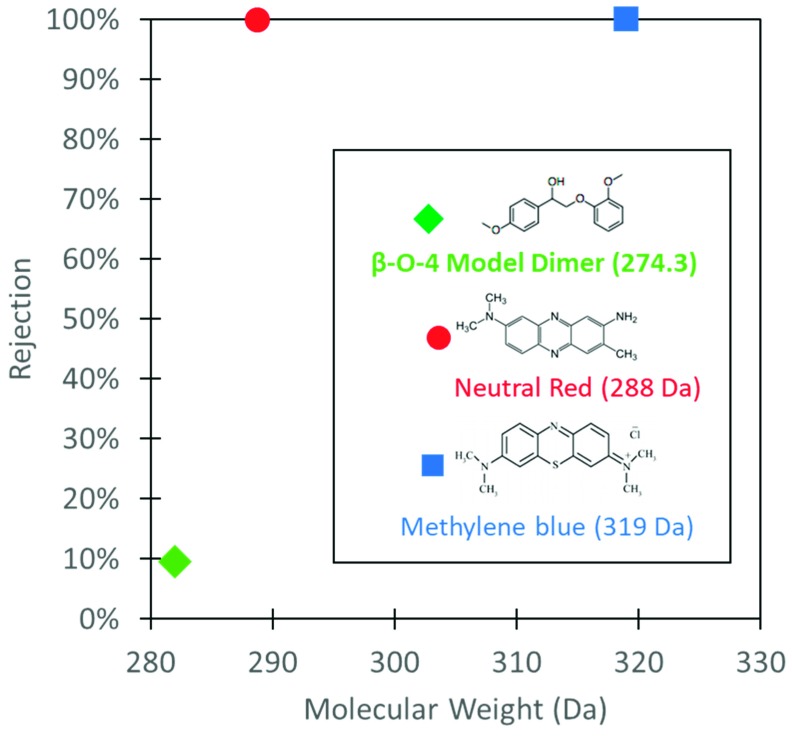
Rejection of model dyes and molecules in iron cellulose composite membranes.

Rejection studies with model dyes also suggests other factors contribute to solute selectivity other than size exclusion. Selectivity vs. molecular weight for small model molecules is show in Figure 6. Rejection of β-O-4 Model Dimer was only 10% despite the MW only being 7 Da less than neutral red. The disparity in rejection can be attributed to interaction among the hydrophilic functional groups. The positive dipoles of the amine groups in the dyes interacts more strongly with negative dipoles of hydroxy groups in cellulose reducing rate of diffusion of the dyes through the membrane. Carboxyl groups in the model dimer do not react as strongly. Rotational freedom in the model dimer may also allow for the dimer to change confirmation as it moves through the membrane, thus increasing diffusion rate. Ring structures in the model dyes prevent rotation within dyes as they move into the membrane domain. Interaction among functional groups and molecular structure must be considered when evaluating possible application of nanofiltration for small molecule separation.

### 3.3. Polyacrylic Acid Cellulose Composite Membranes

**Figure 7:**
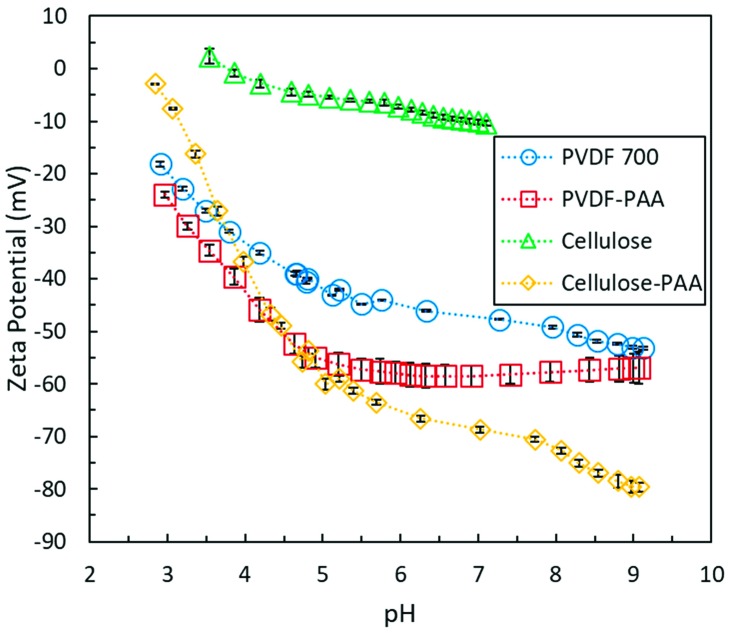
Zeta potential of cellulose (10 wt% cellulose in casting solution)^1^, and cellulose- polyacrylic acid (PAA) membranes in the pH range of 3–9. PVDF 700 membrane obtained from Solecta Membrane and PVDF-PAA (weight gain of 7.28% with functionalization) were synthesized for this study following the procedure established in Islam et al. as a control system to demonstrate the impact of PAA functionalization on membrane surface charge [17].

Polyacrylic acid (PAA) has many negatively charged carboxyl groups which can be utilized for pH responsive behavior, metal capture, and rejection of negatively charged ions. PAA disperses fully in the ionic liquid solvent allowing even mixing with cellulose. Entanglement with cellulose chains and hydrogen bonding with cellulose allow for the retention of PAA after phase inversion. The pKa of carboxyl groups was useful in confirming its presence of PAA at the surface of the PAA cellulose composite membranes. Zeta potential analysis (Figure 7) clearly shows that incorporating PAA into cellulose membranes results in a greater magnitude of negative surface charge that corresponds to a pKa shift at pH 3–5, as expected for carboxyl groups. This behavior has been demonstrated in our previous studies with PAA functionalized PVDF (polyvinylidene fluoride) microfiltration membranes. Due to the dissolution of PAA and cellulose together in ionic liquid, it is hypothesized that PAA was also integrated through the depth of the membrane.

**Figure 8:**
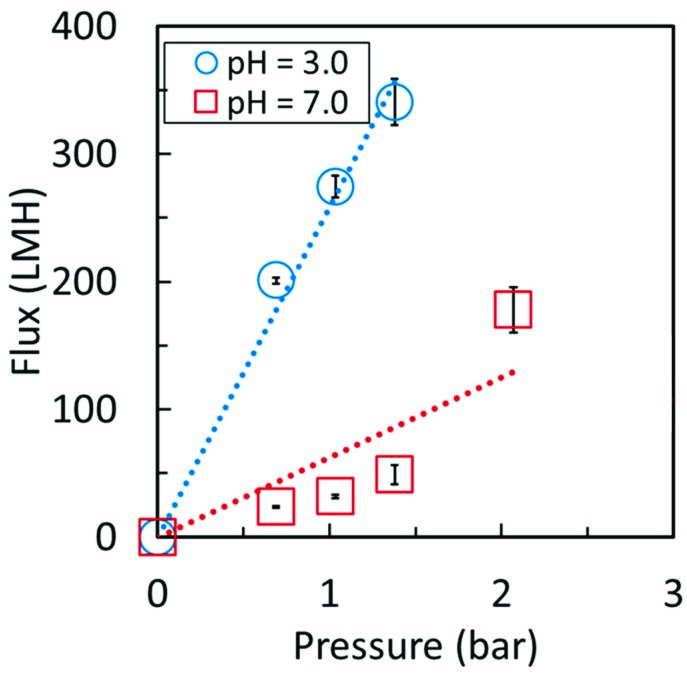
Volumetric flux of deionized water through of cellulose-PAA membrane at pH 3 and 7. Membrane surface area = 13.2 cm^2^.

Further confirmation of PAA in the membrane was necessary to confirm presence beyond the surface. Pressure dependent flux of PAA cellulose membranes were studied at below and above the pKa of PAA. As observed in other PAA functionalized membranes, swelling should occur as carboxyl groups are charged when pH increases above 3. Figure 8 shows the pH responsive behavior of the functionalized membrane. The four-fold decrease in flux when transitioning to pH 7 from pH 3 strongly suggests presence of PAA throughout the entire selective layer of the composite membrane. At high pH the swollen PAA creates a selective layer capable of rejecting 44% of 5kDa blue dextran, while at low pH the PAA collapses, opening the membrane pores.

**Figure 9:**
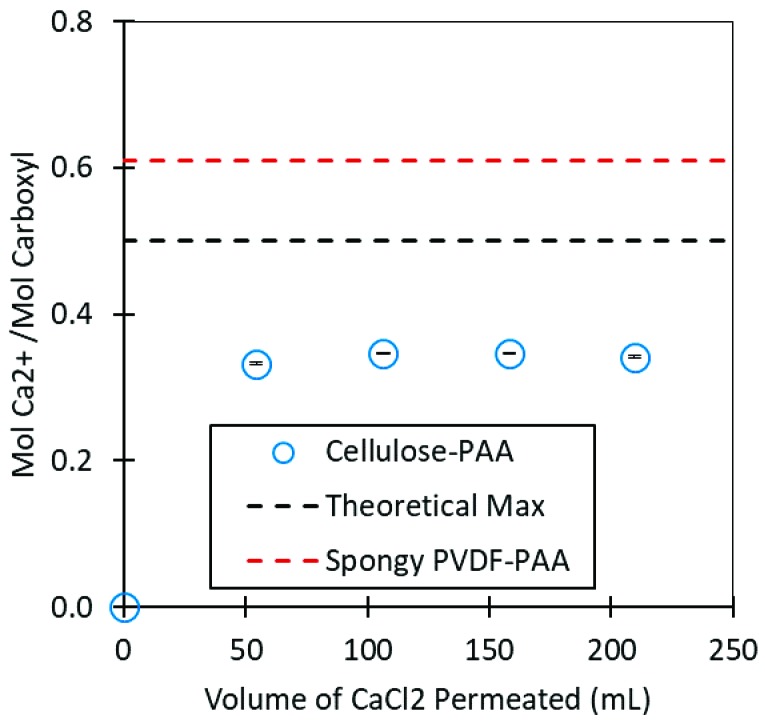
Total Ca^2+^ capture of a 13.2-cm^2^ cellulose-PAA membrane during convective flow of CaCl_2_ (overall flux = 89 LMH and average pressure of 50-mL increments = 0.72 bar) and of a PVDF-PAA membrane from literature after convective flow of CaCl_2_ [23]. Standard deviation was determined via deviation of known samples after spiking with a separate known and thus represents analytical error during inductively coupled plasma optical emission spectroscopy (ICP-OES).

PAA has been utilized for capture of metals due to the ion exchange capacity of the vast network of carboxylic groups. Ion exchange capacity studied for this membrane using Ca^2+^ to better understand the quantity of PAA in the membrane and the accessibility of PAA to ions transporting through. Previous functionalized membrane platforms have not completely answered the question of whether the entirety of the hydrogel is available for ion exchange, or whether channeling occurs within the hydrogel domain. In this scenario PAA is entangled along with the cellulose composite membrane which should theoretically prevent channeling. Ca^2+^ adsorption is shown in Figure 9. This was not observed in cellulose membranes. likely due to the constrained environment in which the PAA is present. The cellulose-PAA membrane demonstrated a maximum Ca^2+^ capture of 0.27 mg, which was equal to 0.055 g Ca^2+^/g membrane and 0.35 mol Ca^2+^/mol carboxyl; this result was confirmed by a mass balance that indicated a 98.2% retention of the fed Ca^2+^ in either the permeate samples, the retentate, or the membrane as well as a difference of 6.6% in measured concentration between the 50 ppm calibration curve sample and the duplicate of this sample. Ca^2+^ capture was roughly 70% of the theoretical maximum and just over half of the 0.61 mol Ca^2+^/mol carboxyl that was calculated for the spongy PVDF-PAA membrane reported in literature [23]. Ca^2+^ capture observed in spongy PVDF-PAA membranes exceeded the theoretical value due to counter ion condensation phenomena within the membrane.

**Figure 10:**
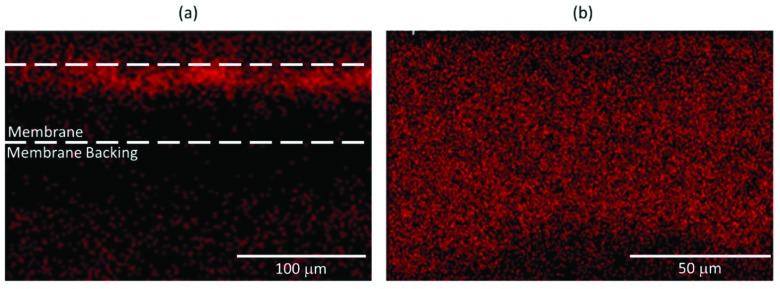
(**a**) Iron energy-dispersive X-ray spectroscopy (EDS) map of the cross section of a PVDF-PAA-Fe sample and (**b**) calcium EDS map of most of the cross section of a cellulose-PAA-Ca^2+^ sample.

Electron dispersive x-ray spectroscopy of the PAA cellulose composite membrane was conducted to determine where metal ion capture was occurring within the membrane. The EDS mapping reveals that PAA cellulose membranes show even dispersion of divalent ions adsorbed throughout the membrane, while PAA functionalized PVDF membranes show divalent ion adsorption only toward the surface of the membrane. The EDS map (Figure 10) serves as further confirmation that PAA is evenly dispersed throughout the membrane.

### 3.4. Lignin Sulfonate Cellulose Composite Membranes

Lignin and cellulose are major constituents of woody plants and interact to create a robust structure that is resistant to decomposition from bacteria and fungi even after the plant’s death. Lignin contains many hydrophilic groups, including phenols which give antibacterial properties [24,25]. Houtman et al. have determined through molecular simulation that hydrophilic groups allow for lignin to adsorb to cellulose microfibrils [26]. Lignin sulfonate, a byproduct of chemical paper pulping industry, is an inexpensive and commercially available source of lignin. The sulfonation process adds hydrophilicity and allows for easy dissolution in 1-ethyl-3-methylimidazolium acetate [27]. Therefore, we sought to use lignin sulfonate as a composite material for cellulose membrane creation. The primary objectives were to determine the effectiveness of the lignin composite membrane and probe antibacterial behavior.

**Figure 11:**
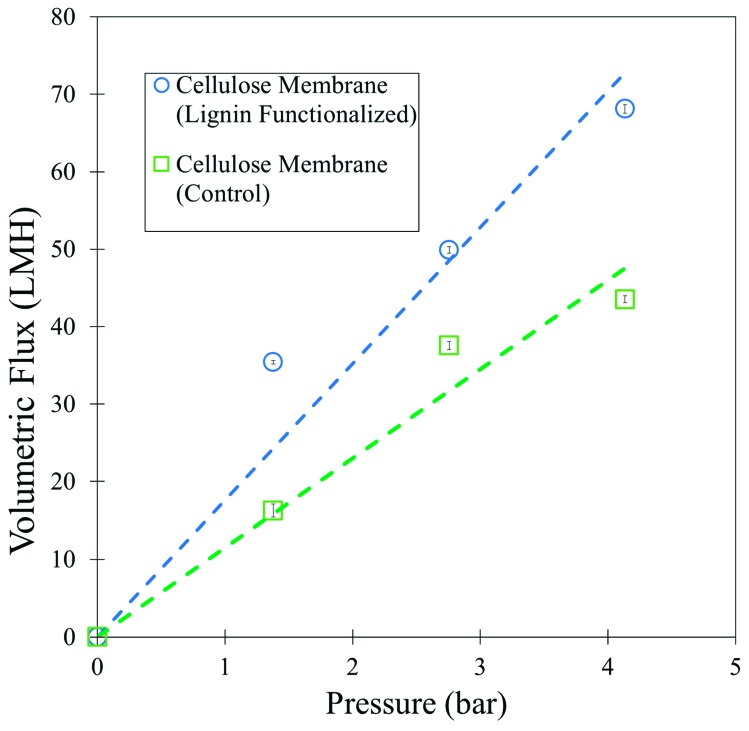
Pressure dependent volumetric water flux through cellulose membranes modified with lignin sulfonate and unmodified cellulose membranes (control).

Water permeability of the lignin cellulose membrane was shown to be roughly double that of the unmodified cellulose membrane (Figure 11). Likely hydrophobic regions of lignin sulfonate cause opening of the selective layer due to poor interaction with cellulose after phase inversion. The viscosity of the dope solutions was particularly high when lignin sulfonate was added as a composite, which may further effect demixing during phase inversion. The rejection of 5000 Da blue dextran was 59%, and this is 16% lower than unmodified cellulose. Neutral red was shown to absorb strongly in within the membrane, which indicates the potential of strong interaction with sulfonate groups within the membrane.

**Figure 12:**
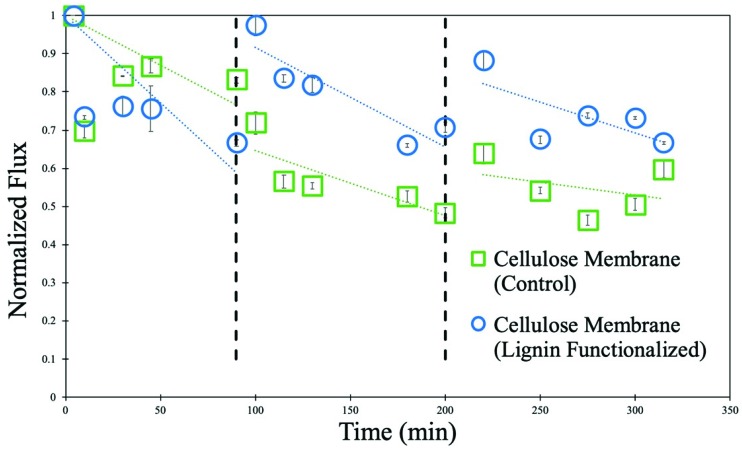
Normalized water flux of cellulose membrane functionalized with lignin sulfonate and of control cellulose membrane during filtration of 100 mg/L humic acid solution. Normalized flux = water flux with humic acid/pure water flux. pH = 5. Operating pressure = 10.4 bar. Vertical dashed lines indicate points during the experiment where tangential washing (1.5 L/min) with deionized ultrafiltered (DIUF) was performed to recover membrane flux. Trend lines (dotted) and flux error bars are shown in the figure.

In order to characterize the fouling of a 10 wt% cellulose in IL membrane functionalized with 2 wt% lignin sulfonate, both a 10 wt% cellulose in IL membrane and a 10 wt% cellulose in IL membrane with 2 wt% lignin sulfonate (cellulose–lignin membrane) were placed in a cross-flow cell. 100 mg/L humic acid solution was passed through the cross-flow cell and the flux of both membranes was recorded over a total of 350 min. The black dotted lines indicate when both membranes were rinsed for 10 min with deionized water (pH of 5.6) to measure the effect of irreversible fouling. The normalized water flux of both the control membrane and the functionalized membrane are contained in Figure 12. The flux of both the control membrane and the flux of the membrane functionalized with lignin sulfonate decreased as more humic acid solution was passed. Error bars for each point indicate high water flux reproducibility. However, after each tangential rinse with deionized water, there is far less irreversible fouling of the functionalized membrane when compared to the control membrane. After only 10 min of rinsing, the functionalized membranes almost completely returned to the initial volumetric flux as recorded before fouling, while the control membrane shows only about 50% recovery of volumetric flux. Although the lignin sulfonate appears to have a negligible effect on the reversible fouling of the membrane, the lignin sulfonate does have a significant effect on reducing the prevalence of irreversible fouling of the functionalized membrane.

**Figure 13:**
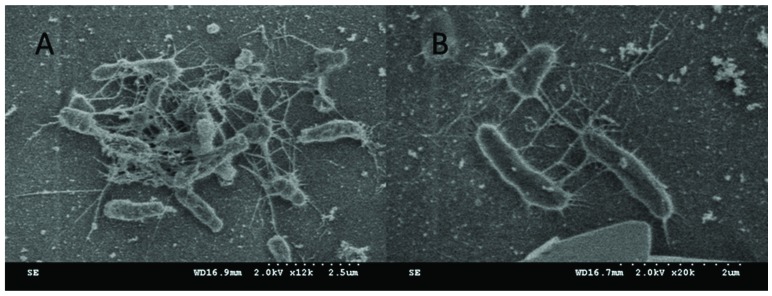
Bacteria growth on (**A**) unmodified cellulose membrane and (**B**) lignin sulfonate modified cellulose membrane.

Beyond natural organic matter and proteins, a key contributor to membrane fouling are biofilms formed by microorganisms that adsorb to the membrane surface. Since, it has been understood for many years that functional phenolic groups provide antimicrobial properties in lignin, the antimicrobial properties of lignin sulfonate modified cellulose membrane was studied [28]. Lignin sulfonate modified cellulose membranes were inoculated with bacteria by filtering a dilute solution of bacteria through the membrane. The bacterial were then given dilute amounts of nutrients and allowed to grow. Bacteria colonies were analyzed after fixation to qualitatively determine the rate of production of extracellular matrix. The SEM images of the membrane surface after bacteria growth can be seen in Figure 13.

### 3.5. Lignin Sulfonate Functionalized Commercial Nanofiltration Membrane

**Figure 14:**
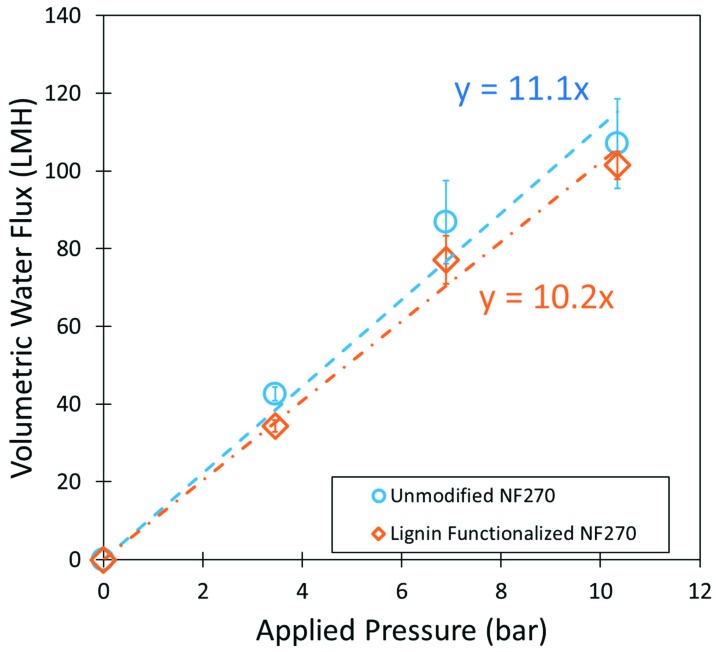
Pressure dependent water flux of unmodified NF270 and LS functionalized membrane.

Lignin sulfonate can also be directly functionalized onto the surface of commercial nanofiltration membranes. Sulfonated lignin has shown potential antifouling properties when deposited onto the surface of thin film composite membranes. This study looked to use heat to esterify lignin to the surface of NF (nanofiltration) membranes. Membrane water permeability was shown to decreases slightly after functionalization (Figure 14), but flux decline was less than 10 wt%. This decline in flux was likely due to the surface functionalized layer adding resistance to flow through the membrane. Lignin has a bulky branching structure that could cause additional hydraulic resistance to flow.

**Figure 15:**
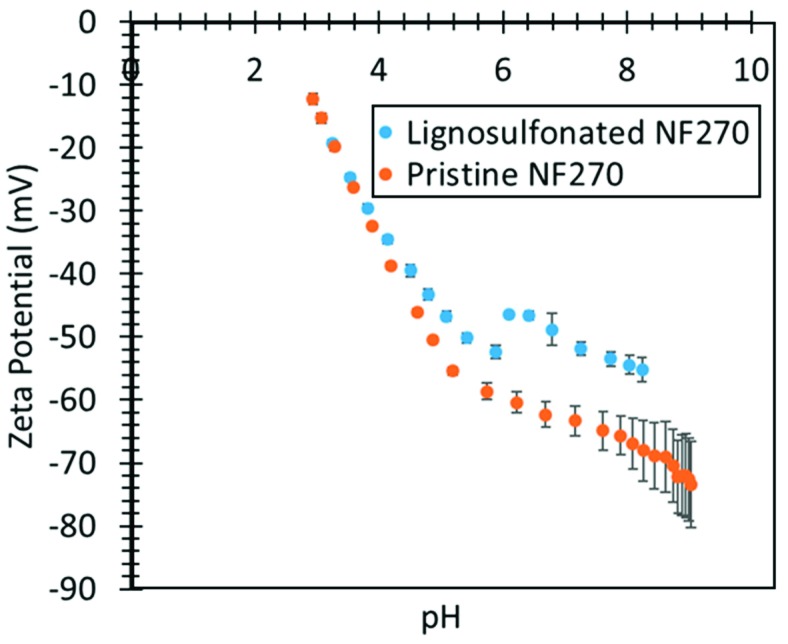
Zeta potential vs. pH for lignin functionalized and pristine NF270 membrane. 100 mg/L KCl used as an electrolyte.

Rejection of Na_2_SO_4_ (1000 mg/L solution) decreased to 97.3% from 98% after functionalization with lignin sulfonate, which is within experimental error for the small samples of membrane tested (20 cm^2^). Zeta potential data also suggests reduction in the number of carboxyl groups on the surface of the NF membrane (Figure 15).

**Figure 16:**
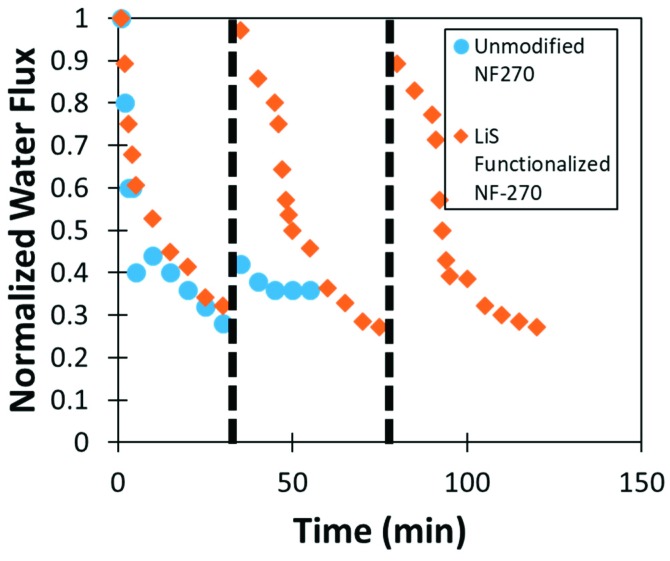
Normalized water flux of lignin sulfonate functionalized and unmodified NF270 during filtration of 100 mg/L BSA. Dotted lines indicate 10 min of tangential rinsing with deionized water (pH = 5.6).

Most excitingly, lignin sulfonate functionalized membranes show promise for use as an antifouling surface. BSA was used as a model foulant and passed through the membrane in cross flow operation. BSA fouling during filtration can be seen in Figure 16. While lignin sulfonate appears to have negligible impact on reversible fouling, irreversible fouling was shown to be far less prevalent after functionalization with lignin sulfonate. Functionalized NF270 membranes showed almost complete recovery of volumetric water flux after just 10 min of tangential flow rinsing with DI water while the unmodified membrane flux only recovered to 40% of the initial value after rinsing. Lignin functionalized NF270 membranes were shown to maintain 90% of the initial flux after the second rinse cycle.

## 4. Conclusions

This study has shown 1-ethyl-3-methyl imidazolium acetate ionic liquid can be utilized as a cosolvent to integrate iron, polyacrylic acid (PAA), or lignin sulfonate with cellulose membrane. Composite and blended materials were found to add unique properties such as pH responsive flux and antibacterial behavior. Performance of iron–cellulose composite membranes demonstrates that composite materials modify membrane structure and impact transport of solvent and solute through the membrane. Membrane structure was observed to become less selective in solvent conditions where affinity between iron and cellulose is reduced. Both steric entrapment and hydrogen bonding allow for PAA to be incorporated into the cellulose membrane domain for hardness ion capture applications. In the same manner, lignin sulfonate was incorporated covalently to reduce irreversible fouling on the membrane surface. This antifouling behavior was also observed when lignin sulfonate was functionalized onto the surface of the commercial NF270 membrane. Ultimately, as ionic liquids continue to be used as solvents for membrane synthesis, composite material should be strongly considered as means to add value or otherwise optimize membranes. Even inexpensive materials such as iron or sulfonated lignin have shown potential as composite materials, and impart little additional costs compared to the price of ionic liquid.

## Figures and Tables

**Table 1:** Model dyes tested for rejection.

Model Solute	Molecular Wt. (Da)	Structure
β-O-4 Model Dimer	282	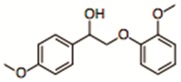
Neutral Red	289	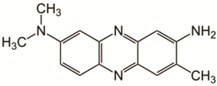
Methylene Blue	320	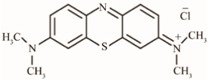

**Table 2:** Composite membranes studied with compositions and relevant properties.

Composite Material	Wt% Composite	Wt% Cellulose	Casting Solution Viscosity (Pa*s)	Water Permeability (LMH/bar) (pH=7)	Rejection (%) 5kDa Blue Dextran
Iron	4	5	6.8	17.4	>99
PAA	2	5	44	267	44
Lignin	5	10	96	17.5	59
Cellulose only	0	10	22.8	9.6	75
